# Laser-Induced Reactions of 4-Aminobenzenthiol Species Adsorbed on Ag, Au, and Cu Plasmonic Structures Followed by SERS Spectroscopy. The Role of Substrate and Excitation Energy – Surface-Complex Photochemistry and Plasmonic Catalysis

**DOI:** 10.1021/acsomega.4c00121

**Published:** 2024-01-25

**Authors:** Ivan Kopal, Marie Švecová, Vojtěch Jeřábek, David Palounek, Tereza Čapková, Alena Michalcová, Ladislav Lapčák, Pavel Matějka, Marcela Dendisová

**Affiliations:** † Department of Physical Chemistry, University of Chemistry and Technology Prague, Technická 5, Prague 6 166 28, Czech Republic; ‡ Institute of Photonics and Electronics, Czech Academy of Sciences, Chaberská 1014/57, Prague 8 182 00, Czech Republic; § Department of Analytical Chemistry, University of Chemistry and Technology Prague, Technická 5, Prague 6 166 28, Czech Republic; ∥ Centre of Polymer Systems, University Institute, Tomas Bata University in Zlín, Třída Tomáše Bati 5678, Zlín 760 01, Czech Republic; ⊥ Department of Metals and Corrosion Engineering, University of Chemistry and Technology Prague, Technická 5, Prague 6 166 28, Czech Republic; # Central Laboratories, University of Chemistry and Technology, Technická 5, Prague 166 28, Czech Republic

## Abstract

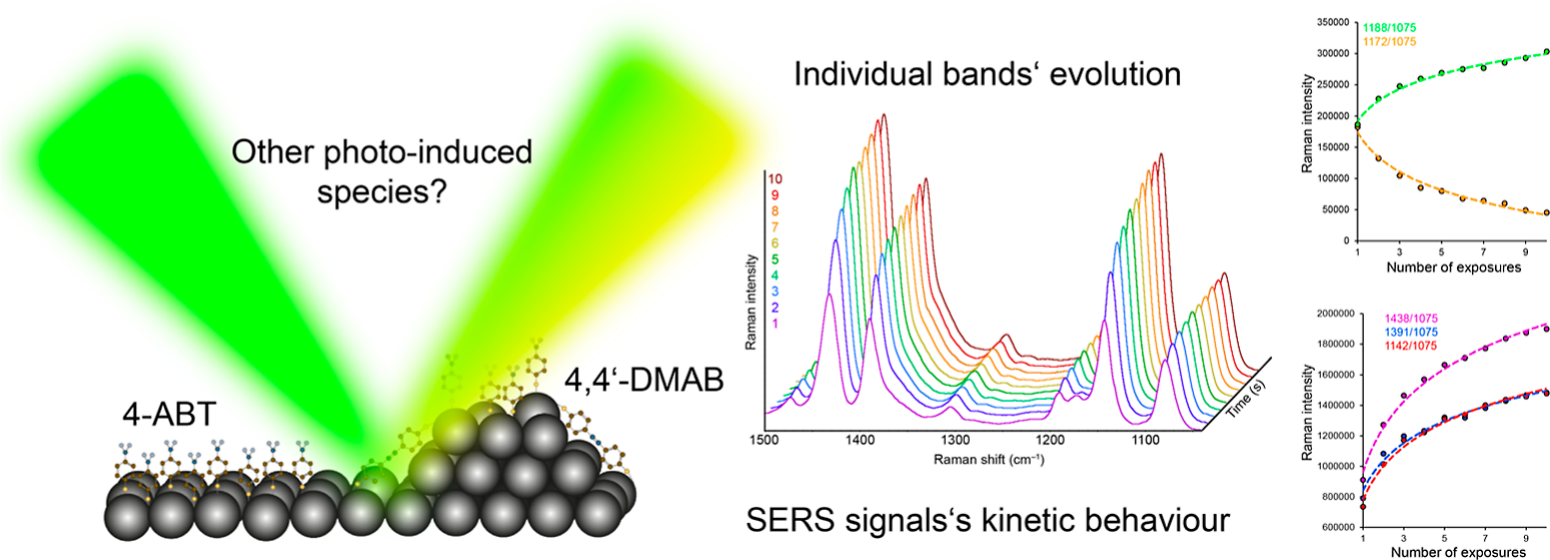

This study focuses on investigating the laser-induced reactions of various surface complexes of 4-aminobenzenethiol on Ag, Au, and Cu surfaces. By utilizing different excitation wavelengths, the distinct behavior of the molecule species on the plasmonic substrates was observed. Density functional theory (DFT) calculations were employed to establish the significant role of chemical enhancement mechanisms in determining the observed behavior. The interaction between 4-aminobenzenethiol (4-ABT) molecules and plasmonic surfaces led to the formation of surface complexes with absorption bands red-shifted into the visible and near-infrared regions. Photochemical transformations were induced by excitation wavelengths from these regions, with the nature of the transformations varying based on the excitation wavelength and the plasmonic metal. Resonance with the electronic absorption transitions of these complexes amplifies surface-enhanced Raman scattering (SERS), enabling the detailed examination of ongoing processes. A kinetic study on the Ag surface revealed processes governed by both first- and second-order kinetics, attributed to the dimerization process and transformation processes of individual molecules interacting with photons or plasmons. The behavior of the molecules was found to be primarily determined by the position and variability of the band between 1170 and 1190 cm^–1^, with the former corresponding to molecules in the monomer state and the latter to dimerized molecules. Notably, laser-induced dimerization occurred most rapidly on the Cu surface, followed by Ag, and least on Au. These findings highlight the influence of plasmonic surfaces on molecular behavior and provide insights into the potential applications of laser-induced reactions for surface analysis and manipulation.

## 1 Introduction

During the decades that have passed since the first observation and successive careful and convoluted discussions about the phenomenon of surface-enhanced Raman scattering (SERS), the technique has become well-established and well-known.^1^ Nowadays, thanks to its dynamic development, it can be found in many scientific branches that deal with the study of issues at the level of individual molecules as well as more and more sophisticated and applicable methods of its use in the field of analytical chemistry.^2,3^ The very fact that various plasmonic substrates are key players in SERS spectroscopy is behind the gradual development of another independent branch strongly related to molecular (“photochemical”) transformations on plasmonic surfaces, most often referred to as “plasmonic catalysis”.^2,4−7^

In the current state of knowledge, several types of chemical reactions are distinguished that can occur on the surfaces of plasmonic structures, depending on the ability of specific substances to absorb the radiation of the used laser, the ability of the prepared nanostructures to scatter/absorb radiation and many other factors.^5^ In conjunction with SERS, which provides us first with information about individual species that are exposed to the incident laser on specific surfaces, we can observe ongoing induced molecular transformations (mainly chemical reactions) in live transmission and, in ideal cases, learn new facts about (i) the physicochemical nature of transformation (reaction mechanisms), (ii) the formation of hitherto unknown side products and transient species,^8−11^ and/or (iii) structural variations of the systems (e.g., surface (dis)ordering accompanied by molecular reorientation and symmetry changes).

One of the substances that seems destined to raise a seemingly never-ending set of questions about its behavior on plasmonic surfaces is 4-aminobenzenethiol (4-ABT).^8,12−18^ Spectral bands of 4-ABT often observed in the SERS spectra could not be assigned to this molecule based on the knowledge of its normal Raman spectra.^18^ The origin of these bands was initially attributed to the enormous action of the charge transfer (CT) mechanism.^19^ Although due to the very suitably positioned levels of the molecular orbitals, a significant effect of the CT mechanism is very likely,^20^ further studies of this molecule proved that dimerization of individual molecules can occur as a result of the impact of radiation.^13,21^ The newly formed compound is known under the name “4,4′-dimercaptoazobenzene” (4,4′-DMAB), and based on DFT simulations, it is possible to declare that the discussed bands belong to the dimerized molecule.^14^ Despite the considerable number of studies that have dealt with both 4-ABT and 4,4′-DMAB, many questions remain unanswered. The collected spectra often differ, as does more detailed information about the reaction mechanism.^22−25^ Another investigation of these molecules deserves more attention not only for a principal understanding of the transformation mechanism but also in terms of the fact that 4-ABT is a popular model molecular probe for testing the potential enhancing abilities of newly prepared substrates.^26−29^

In this study, we performed experiments on Ag, Au, and Cu plasmonic surfaces to assess the catalytic properties and interaction modes of the molecules depending on the metal used. The structure and plasmonic capabilities of the substrates were investigated in detail by electron microscopy and visible (Vis) spectroscopy. A total of five excitation wavelengths (455, 532, 633, 780, and 1064 nm) were used to obtain exact information regarding the kinetics and the extent of the reaction at different photon energies, which cover a wide range of excitation wavelengths used in SERS spectroscopy. The recorded data were carefully interpreted using the DFT calculations and peak resolve function, thanks to which information regarding the orientation of the molecules to the surface, the interaction with the surface, and the formation or disappearance of individual 4-ABT species (ABTS) during subexperiments were obtained. Moreover, it was possible to focus on the area of chemical kinetics of the investigated reaction and obtain information on the order of the reaction and the rough value of the rate constant.

## 2 Experimental Section

### 2.1 Materials

The following chemicals were used in the study: 4-aminobenzenethiol (Fluka, 95.00%), ammonia (Lachner, 26.00%), silver nitrate (Sigma-Aldrich, 99.99%), sodium hydroxide (Penta, 98.00%), ammonium chloride (Penta, 99.50%), copper(II) chloride (Sigma-Aldrich, 97.00%), sulfuric acid (Lachner, 96.00%), tetrachloroauric acid (Sigma-Aldrich, 99.99%), methanol (Lachner, 99.95%), aluminum oxide (Sigma-Aldrich, 99.50%), hydrogen peroxide (Penta, 30.00%), and calcium carbonate (Lachner, 99.00%). All chemicals were used in the form supplied by the manufacturer and were not further purified. In the preparation of aqueous solutions, Milli-Q water was used, prepared using the Millipore system. 4-ABT was dissolved in methanol for deposition bath preparation purposes.

### 2.2 Nanostructured Substrate Preparation

Large-scale Ag, Au, and Cu SERS substrates prepared by electrodeposition on platinum targets were used in this work.^3^ Pt targets were first polished with metallographic papers, polished with Al_2_O_3_ and CaCO_3_, and cleaned by immersion in a mixture of H_2_SO_4_ and H_2_O_2_ in a volume ratio of 3:1 for 30 min, after which they were rinsed with Milli-Q water and dried. Then, the targets were electrocoated using different current sequences in the respective electrochemical baths (Table 1). The prepared substrates were subsequently immersed into the 4-ABT deposition bath (with a base concentration of 10^–4^ mol·dm^–3^), in which they were kept for 24 h. Subsequently, the prepared samples were removed from the solutions, rinsed with methanol, and dried in a stream of nitrogen. The prepared samples were subsequently stored in PE sample boxes without any other treatment until the time of implementation of experiments.

**Table 1:** Used Baths and Current Sequences of Plate Platinum Targets

[Ag(NH_3_)_2_]Cl	*I* (mA)	5	10		
	*t* (min)	10	5		
[Au(NH_3_)_4_]Cl_3_	*I* (mA)	5	10	15	
	*t* (min)	5	5	5	
[Cu(NH_3_)_4_]Cl_2_	*I* (mA)	5	10	15	20
	*t* (min)	5	5	5	5

### 2.3 Electron Microscopy

Nanostructured surfaces were characterized by electron microscopic methods (Scanning Electron Microscopy − SEM and Transmission Electron Microscopy – TEM). A VEGA 3 LMU scanning microscope (TESCAN, Czechia) was used for initial information about the surface. The integrated energy-dispersive X-ray spectroscopy (EDS) analyzer INCA 350 (OXFORD Instruments, Great Britain) enables the simultaneous study of morphology and the chemical composition of the prepared substrate. The transmission microscope – EFTEM 2200 FS (Jeol, Japan) – was employed for a more detailed examination of the structures. The surface layer was wiped with a cotton swab, and then the adhered nanostructures were transferred to isopropanol. The sample thus prepared was then stretched on a 300-mesh lacey carbon TEM grid, which was subsequently introduced into the microscope. This microscope is equipped with an EDS analyzer that works with a spectral resolution of 1–2.4 nm.

### 2.4 Vis Spectroscopy

Vis absorption spectra of 4-ABT solution were acquired using a CARY 50 spectrometer (Varian, Australia), which operates in a range of wavelengths from 190 to 1100 nm. The spectra were collected with a set scanning speed of 360 nm·min^–1^ and a 1.5 nm resolution in the range from 300 to 800 nm. As a radiation source, the spectrometer uses a xenon discharge lamp working in pulse mode. The analysis of the 4-ABT methanolic solution with a concentration of 10^–3^ mol dm^–3^ took place in a 5 mm quartz cuvette. The diffuse reflectance (contributed with absorption, reflectivity, and scattering effects) spectra of the plasmonic targets were accumulated with a Shimadzu UV-2700 dual-beam spectro-meter (Shimadzu Corp., Japan) in a diffuse-reflective arrangement using a 60 mm integrating sphere. The spectra were obtained in the range of 300–800 nm with a 0.2 nm step.

### 2.5 SERS Spectroscopy

SERS spectra were obtained with three excitation wavelengths from the visible region (455, 532, and 633 nm) and two from the near-infrared (NIR) region (780 and 1064 nm). To accumulate SERS spectra with a NIR excitation of 1064 nm, an infrared spectrometer with Fourier transformation (FT-spectrometer) EQUINOX 55 (Bruker, Germany) connected to the FRA 106/S Raman module was employed. A dispersive Raman spectrometer DXR Raman microscope (Nicolet, USA) was used for the acquisition in the visible and NIR range. It uses four exchangeable radiation sources. For radiation emission of various wavelengths, diode lasers (455 and 780 nm), a Nd:YAG (Neodymium-Doped Yttrium Aluminum Garnet) laser excited by laser diodes (532 nm), or a He–Ne gas laser (633 nm) are utilized. Scattered radiation is detected by a charge-coupled device multichannel detector. The acquisition parameters for both devices are listed in Table 2.

**Table 2:** Acquisition Parameters of Individual SERS Spectra of ABTS on Different Surfaces

	excitation wavelength (nm)	mean resolution (cm^–1^)	metal	laser power (mW)	expositions	time (s)
FT-spectrometer	1064	4	Ag	300	1024	—
			Au	300	1024	—
			Cu	300	1024	—
dispersive spectrometer	780	4.4	Ag	1	20	1
			Au	1	20	5
			Cu	1	20	1
	633	4.4	Ag	1	20	1
			Au	1	20	1
			Cu	1	20	1
	532	4.4	Ag	0.5	20	1
			Au	1	20	5
			Cu	1	20	1
	455	4.4	Ag	1	20	1
			Au	1	20	5
			Cu	—	—	—

**Figure 1:**
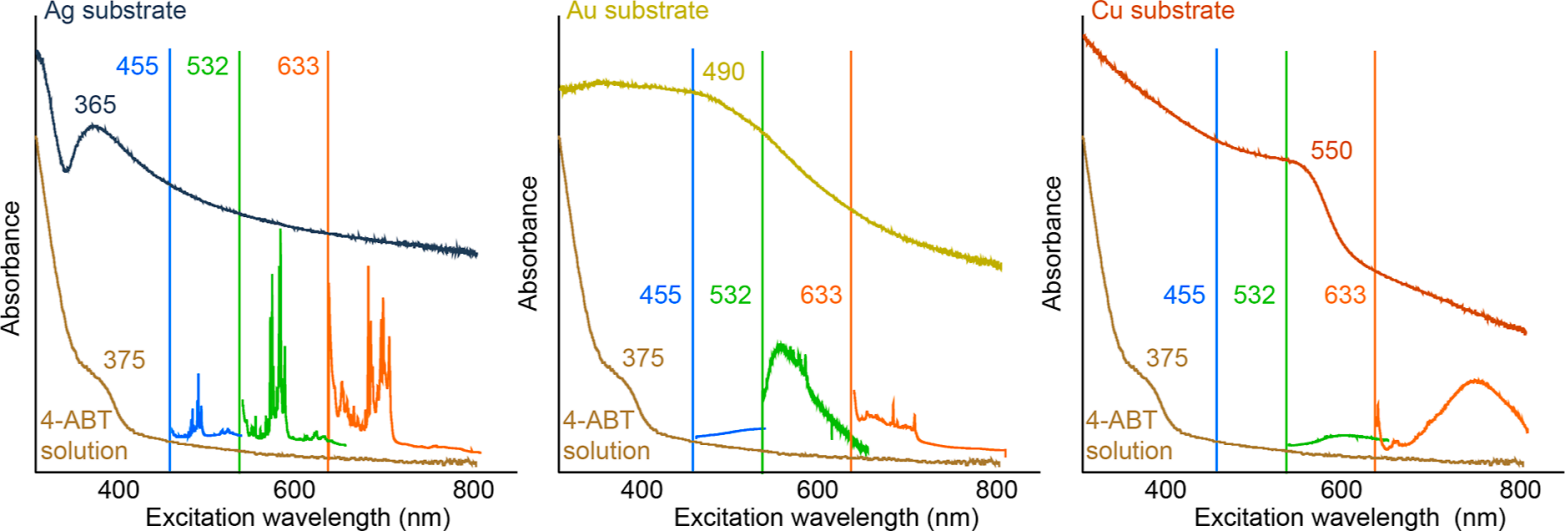
Schematic representation of the positions and shape of the SPR spectra of individual substrates including the absorption band of 4-ABT (methanolic solution, 10^–3^ mol·dm^–3^), selected excitation laser lines, and corresponding Raman spectra.

All measurements were done with the presence of air in the cuvette space, and no additional gaseous reagent was used to control exact extent of the reactions. Ten spectra (from ten spots) were collected from each substrate and subsequently processed (baseline correction for Au and Cu substrates, averaging where noted in the caption). Spectra presented in Figure 1 were not baseline corrected to emphasize its nature, which is crucial for the further discussion. For kinetic measurements, ten consecutive acquisitions from one place of the substrate were performed. For spectral processing, Omnic 9 software was used. For data evaluation, the band resolution function was used in the spectra, while the spectral bands were fitted by using Voigt profile functions.

### 2.6 DFT Calculations

All DFT calculations were performed using the B3LYP functional and the LanL2DZ basis set using Gausian 16W software. First, a geometry optimization calculation was performed for each type of complex, and then, their Vis and SERS spectra were calculated for the obtained optimal geometries. For all computations, a single metal atom was used to simulate just the surface interaction of the metal atoms with the considered molecules. The shown DFT spectra are scaled by a factor of 0.975.

### 2.7 Kinetic Model Fitting

Time-dependent experimental data of (normalized) SERS intensities were used for the kinetic analysis of the system behavior. The fitting procedure was performed using Maple 2020 software. The “NonlinearFit” function built into the software was used for this purpose. First, the general relation of the form
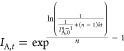
was used. In this equation, *t* denotes time, *n* is the order of the reaction, *I*_A,*t*_ is the SERS intensity at any given time, and *I*_A,0_ is the SERS intensity at *t* = 0. This equation is simply derived from the basic chemical kinetics,^30^ where substance concentration is substituted by SERS intensity in our case. For clarity, we use the term “experimental fit“ as the description for the procedure in this article. For the normalized values of the growing bands (see Section 3.7), the fit based on integrated rate equations for the first- and second-order kinetics was used.^30^

## 3 Results and Discussion

### 3.1 Substrate Characterization

Ag, Au, and Cu substrates were characterized by SEM and TEM including EDS analysis coupled with both of the mentioned techniques. Briefly, while the Ag and Au substrates showed a dendritic morphology (less jagged in the case of Ag and more “fractal-like” in the case of Au), a spherical morphology was noted on the surface in the case of the Cu substrate. EDS analysis showed that the prepared surfaces were chemically uniform, as expected. For more detailed information and records from the TEM and SEM characterization of the substrates, we refer to the Supporting Information (SI, Figure S1–6).

### 3.2 Absorption and Raman Spectroscopy in the Visible Region

Figure 1 shows the shape and positions of the bands of the localized surface plasmon resonance (LSPR) and the absorption spectrum of 4-ABT in a methanolic solution relative to the positions of the individual excitation lines. The overall form of spectral records obtained at different excitation wavelengths from the visible region is also schematically indicated in Figure 1. In the case of the Ag substrate, where the LSPR maximum was at 365 nm, a strong SERS response was achieved for all three excitation wavelengths. From the point of view of the LSPR maximum position, all the used excitation wavelengths 455, 532, and 633 nm (note: also 780 and 1064 nm, which are not marked in the figure because of limited measuring range of Vis spectrometer) have a longer wavelength than the LSPR. The fact that the highest signal intensity was achieved when the excitation wavelengths of 532 and 633 nm were used, which are located further from the LSPR than the wavelength of 455 nm, and a stronger signal could be expected for the shortest excitation line, deserves attention. The course of the LSPR shows that it decreases only very slowly toward higher wavelengths, which may be the reason for the SERS activity even when excitation wavelengths from the NIR region are used (will be shown later).

For the Au substrate, the LSPR maximum was observed at ca. 490 nm, which is noticeably lower than the more commonly observed maximum for most colloidal systems.^31^ The intensities of the SERS bands are also relatively low. The intensity of the LSPR spectrum toward NIR wavelengths again decreases relatively slowly, which corresponds to the fact that even in the case of the Au substrate, the signal was also observed when excitation wavelengths from this region were used. When wavelengths from the visible light region were used, different results were obtained. Spectra obtained at wavelengths of 455 and 532 nm were characterized by a considerably huge background, although in the case of the latter wavelength, the SERS bands of ABTS were clearly discernible. The best Raman band response was observed for the 633 nm wavelength.

The last investigated substrate was Cu and in cases where the SERS response of ABTS was observed, a strong level of “fluorescence-like” background was found, which, especially in the case of the excitation wavelength of 633 nm, does not correspond to the expected position of fluorescence from 4-ABT molecules. The LSPR maximum of Cu substrate is located at ca. 550 nm, which is in very good agreement with colloidal systems measured elsewhere.^32,33^ The excitation wavelengths of 455 and 532 nm are both below the LSPR maximum. However, while the SERS signal was observable (after baseline correction) at the 532 nm wavelength, it was not detectable at the 455 nm excitation. The highest SERS intensity was again detected for an excitation wavelength of 633 nm (after baseline correction). It is obvious that the value of the extinction record of the Cu substrate rapidly decreases toward NIR wavelengths, which suggests that no SERS signal was observed when using excitation wavelengths in this region.

Given the position of the absorption maximum of 4-ABT molecules (325 nm), it cannot be expected that molecular resonance phenomena (of an isolated molecule) could occur in the case of any laser excitation wavelength used.

### 3.3 DFT Calculations of Vis and SERS Spectra

To consider different ways of interaction of the studied molecules with the prepared surfaces and various emerging photochemical products, a series of DFT calculations was performed. Theoretical calculations were aimed at obtaining theoretical Vis and SERS spectra for various “metal-molecule” complexes. In total, we considered six possible molecules/conformations, based on the literature published so far, and implemented our own DFT calculations. In addition to the 4-ABT molecule, we also performed calculations for 4-nitrobenzenethiol (NBT), 4,4′-dimercaptohydroazobenzene (DMHAB), and three different conformations of DMAB (marked as 1–3, their molecular structures are included in the Supporting Information as Figure S7).

**Figure 2:**
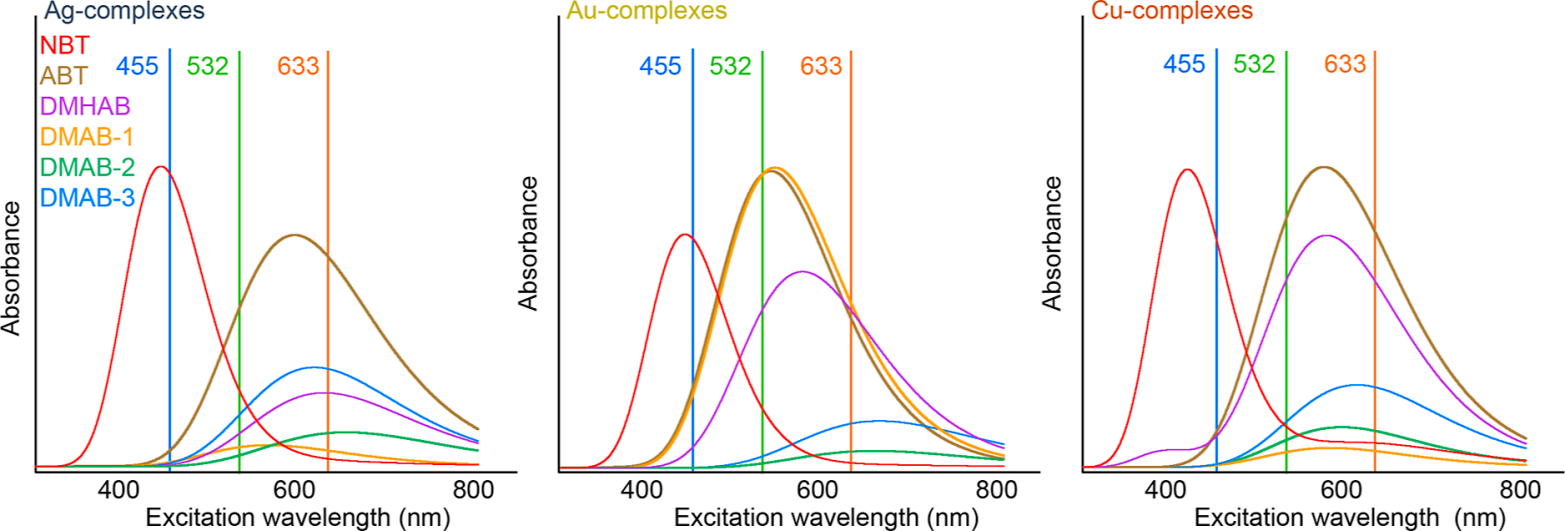
DFT-calculated Vis spectra of the theoretically formed “metal–ABTS” complexes concerning the positions of the excitation lines.

Figure 2 shows the calculated Vis spectra of corresponding metal complexes for the introduced molecules, together with the positions of the excitation lines marked. It is obvious that for all three metals, the band corresponding to the M-NBT (M–metal) complexes has the lowest wavelength position (the highest transition energy), the maximum of which lies approximately in the region of the first used excitation wavelength (in the case of Cu, this position is slightly lower). The band corresponding to the metal complexes with 4-ABT has a maximum between the excitation wavelengths of 532 and 633 nm for all three investigated metals, which are also the wavelengths with the highest recorded SERS response for all three substrates. The maxima corresponding to the complexes with DMHAB and DMAB are correspondingly found in the same region, but their exact positions and intensities differ depending on the applied metal. These bands then often extend up to the region of the 780 nm excitation wavelength, while the bands belonging to Au-complexes extend most into this region. The highest intensity of the DMAB-1 isomer was calculated for the Au substrate, contrary to weak absorption bands for Ag and Cu substrates. However, it was determined through calculations that DMAB-1 is the optimal (the lowest energy state) geometry for the Au substrate, while DMAB-3 was determined to be optimal for Ag and DMAB-2 for Cu.

The positions of the Vis bands of the selected molecules provide valuable information regarding the observation of the fluorescence background. For example, the bands belonging to the complexes with NBT would correspond well to the surface-enhanced fluorescence in the wavelength regions of 532 and 633 nm. At the same time, the band positions of the other molecules would be a good reason for observing the highest SERS intensity at the excitation wavelength for almost all three metals used. This is because selective CT (molecular) resonances could occur in this area because of which the final signal could be amplified by a so-called chemical mechanism.

**Figure 3:**
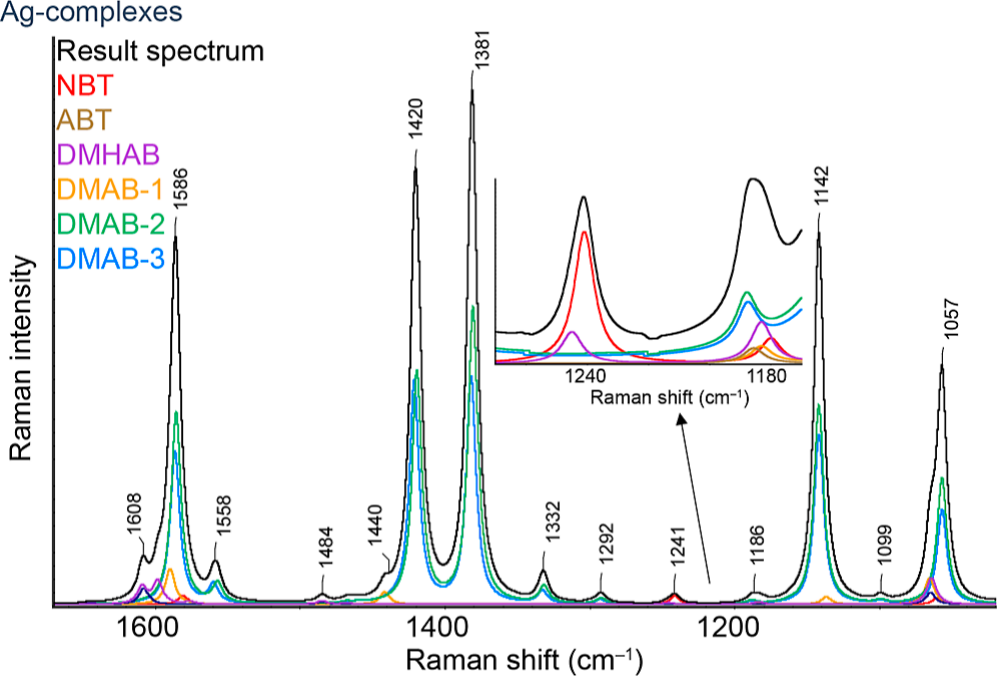
DFT-calculated Raman spectra of “Ag-ABTS” complexes and their theoretical sum spectrum (black line).

Theoretical Raman spectra for Ag are shown in Figure 3. In addition to the spectra corresponding to the individual molecular species, the figure contains the total spectrum of all components (for the theoretical equimolar case, black line), which serves mainly to determine spectrally significant areas worthy of attention during the actual analysis of the experimentally obtained spectra.

Around 1057 cm^–1^, it is possible to observe a group of bands, all of which belong to the C–S stretching vibrations; however, depending on the different types of chemisorbed molecules, their intensity and position shift noticeably (the bands of DMAB are lower than the bands of the other considered molecules). Bands in this region can probably be used with difficulty to distinguish the individual forms of molecules or to assess the rate of the ongoing reaction. On the other hand, due to its considerable “averaging”, this band offers the possibility of using it to relate other bands to the total area of the C–S stretching vibration bands, i.e., use it for basic/rough normalization, which will allow at least a simplified (semi)quantitative analysis of individual ABTS bands.

The band at 1142 cm^–1^ clearly belongs to DMAB molecules, although even here, it can consist of several bands or shoulders depending on the nature of the specific complex. The same can be said about the bands at 1332, 1381, and 1420 cm^–1^, all of which, with respect to the certain range in which other DMAB complexes occur, can be undoubtedly assigned to DMAB molecular complexes. The band at 1441 cm^–1^ should also belong to DMAB, but in this case, it corresponds to its highly symmetrical modification, which is surprisingly characterized by the complete absence of the typically observed bands around 1381 and 1420 cm^–1^, which are commonly used to identify DMAB. Bands at around 1180 and 1240 cm^–1^ exhibit a wide range of characteristics. The bands at around 1180 cm^–1^ are present in all of the considered molecules, but their exact positions differ noticeably. These bands can be separated from each other by using band resolution, allowing for the observation of their behavior, such as their dependence on the excitation wavelength.

The band at 1240 cm^–1^ is characteristic of NBT and DMHAB molecules, with the former showing a relatively intense and well-recognized band. The peaks around 1600 cm^–1^ again belong to all the investigated molecules because all these molecules contain at least one aromatic ring in their structure, for which the in-plane (“stretching”) vibration is typical in this region. These bands are theoretically more suitable for standardization purposes, but with appropriate peak resolution, they could also be used to assess the presence and progress of individual processes.

It is worth noting that similar behavior can be attributed to the other two metals, namely, Au and Cu, based on calculations. It seems that in the case of the same representation of the metal atoms and the orientation between them and the concerning molecules the selected metal almost does not manifest itself in the Raman band position. Of course, it is necessary to take into account the fact that our calculations do not include the effect of surface selection rules, and the assumption of the same number of interacting atoms is highly unrealistic not only for all three surfaces in relation to each other but also with the highest probability for different places on the prepared surface, which will undoubtedly be reflected in the obtained spectral profiles. Even so, the calculated spectra can be used to select significant spectral ranges and to demonstrate the deformation of individual bands due to the presence of various ABTS near the point of incidence.

Due to the considerable similarity of the trends, the calculated spectra for Au and Cu are given in the Supporting Information (Figure S8–9, as well as tables listing the bands and their assignment).

### 3.4 Silver Surface-Enhanced Raman Scattering

**Figure 4:**
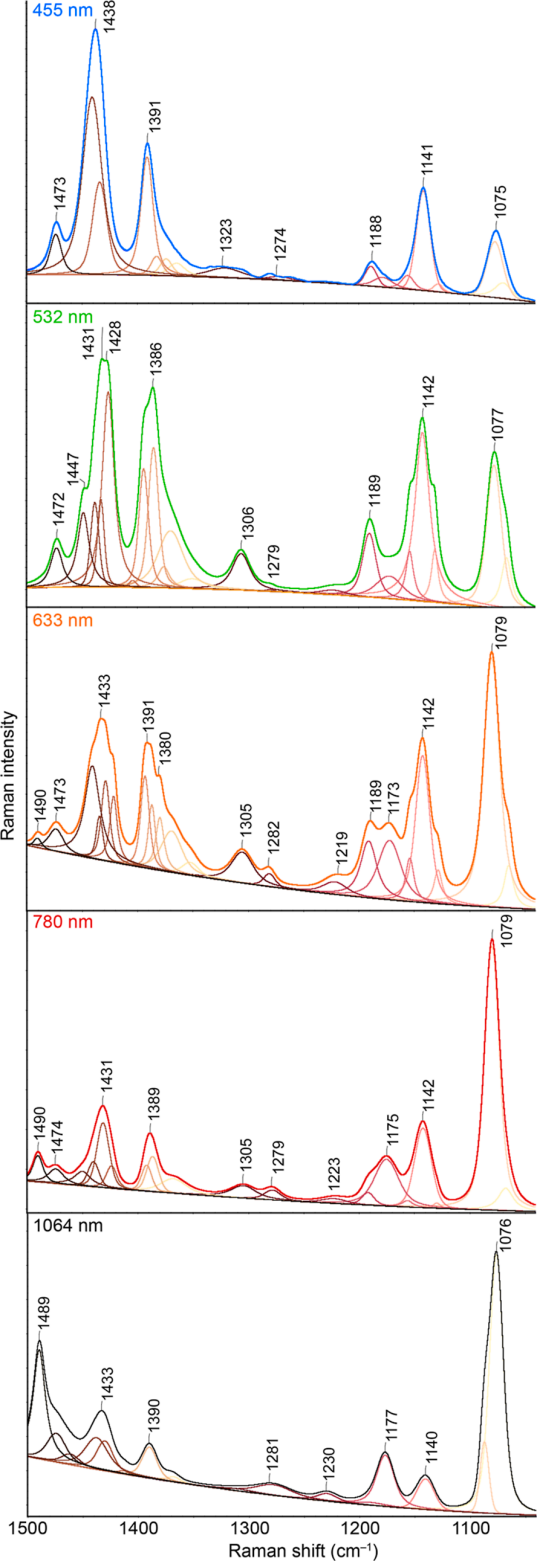
Ag-SERS spectra recorded at the indicated excitation wavelengths and the results of the peak resolve process. Spectra are shown in the full-scale mode.

Selected representative Ag-SERS spectra of ABTS recorded with different excitation wavelengths are compared in Figure 4. Moreover, the partition of the spectra into individual peaks is shown, which was obtained using the peak resolve function in Omnic software. From Figure 4, it is evident at first glance that the profiles of the recorded spectra differ significantly from each other with different excitation wavelengths used.

In all of the mentioned spectra, it is possible to observe a varying degree of occurrence of “alien bands” belonging to some of the molecules from the numerous ABTS family. When all wavelengths are used, for example, there is a band in the vicinity of 1140 cm^–1^ in the spectra, the position of which is also relatively constant within the collected sets of spectra (it varies in the range of 2 cm^–1^). The bands with maxima between 1390 and 1440 cm^–1^ exhibit significant variability in terms of both the position of their maxima and the relative intensity ratios between them. The reason may be that these bands belong to the vibrations of the N=N bond, which is located in the middle of the molecule and thus is strongly influenced by the symmetry and orientation of both thiol groups concerning the atoms of the metallic (plasmonic) surface.

The broadening of bands and abundant occurrence of shoulders are clearly visible, which could tentatively be attributed to the wide range of possibilities for the interaction of reactant and product molecules with the Ag surface mentioned previously. We can observe the most shoulders in the SERS spectra using the excitation wavelengths of 532 and 633 nm, which correlate with the highest signal achieved at these excitation wavelengths. To a certain extent, this finding also supports the idea of molecular resonances with “surface-complex” (CT) transitions in the formed “metal-molecule” complexes, whose absorption bands should be located in this region. For example, at the indicated wavelengths, not only the bifurcation of the band maxima between 1390 and 1440 cm^–1^, but even the predicted shoulder above 1440 cm^–1^, which could belong to a highly symmetric binding variant of the DMAB molecule (referred to as DMAB-1), is evidently visible.

Regarding NBT and DMHAB molecules, they could not be demonstrably found. In the spectra measured with excitation wavelengths of 633, 785, and 1064 nm, however, there is a relatively low-intensity band in the vicinity of 1230 cm^–1^, which could be assigned only to one of these molecules and not any other (based on DFT calculations). It is thus possible, for example, that NBT molecules are an intermediate product of the reaction of 4-ABT to DMAB, or that the energy of these wavelengths better corresponds to the transition to NBT (the wavelength of the maximum of the calculated absorption band of NBT is, however, located relatively low).

**Figure 5:**
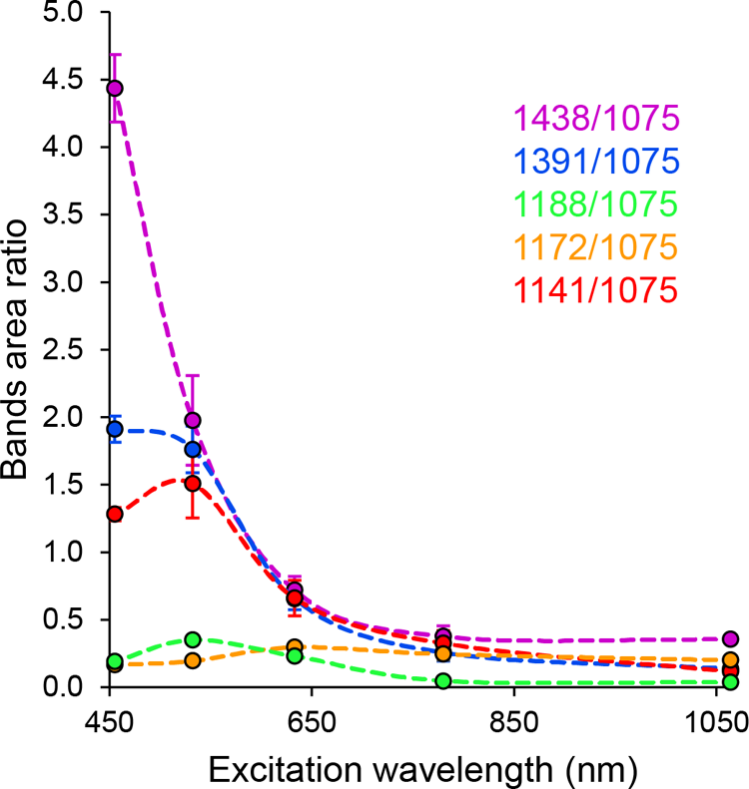
Dependence of the area ratio of the selected Ag-SERS bands on the excitation wavelength.

The behavior of a pair of bands with the maxima at ca. 1170 and 1190 cm^–1^ is also noteworthy. It is evident that while the occurrence of the second-named bands predominates at shorter wavelengths, the first-named band dominates toward the low-energy near-infrared region. In general, it can be stated that the DMAB bands have a higher intensity in the spectra obtained at shorter excitation wavelengths. In the case of NIR wavelengths, they are already noticeably less prominent. The dependence of the ratio of the areas of the noticeable bands to the 1075 cm^–1^ band (for the selection of this “reference” band see above) is compared in Figure 5.

Figure 5 shows the trend indicated in the previous paragraph. In general, the intensities of bands at 1438, 1391, 1188, and 1142 cm^–1^ also decrease with increasing excitation wavelength (decreasing photon energy), while the rather opposite trend applies to band 1172 cm^–1^. It can also be seen from Figure 5 that, for example, the trend for bands 1438, 1391, and 1142 cm^–1^ is very similar for wavelengths longer than ca. 530 nm (even from the point of view of the values of the band area ratios not only from the point of view of the course). However, this statement is contradicted by the values obtained for the shortest examined excitation wavelength of 455 nm, in which case the band ratio value of 1142 cm^–1^ is even lower than that at the less energetic wavelength of 532 nm. The reason is hypothesized by the formation of “metal-molecule” complexes, as a result of which the maxima of the absorption bands of the corresponding molecules would be red-shifted. As a result, upon excitation by 455 nm radiation, a photochemical reaction could occur, the mechanism of which, due to the good agreement of the incident radiation with the excitation energy of the molecule, would differ at least in its course from all other cases under study. The considerably different ratios of the DMAB bands compared to the other excitation wavelengths suggest that molecules with a different preferential orientation may have been formed in this special case.

The behavior of the remaining pair of bands is slightly different. It appears that relative to the area of the band at 1075 cm^–1^, the band at 1188 cm^–1^ reaches its maximum at the excitation wavelength 532 nm; complementary to this, the area of the 1172 cm^–1^ band is the lowest of the monitored interval of excitation wavelengths. In addition, it seems the behavior of the latter band that at a 633 nm excitation wavelength and higher, its ratio to the 1075 cm^–1^ band varies only very slightly depending on the wavelength. At the same time, it is noticeable that at the 532 nm excitation wavelength, the value of the sampling standard deviation of the ratios reaches the highest values for the bands normally attributed to DMAB (1438, 1391, and 1142 cm^–1^). The anomalous behavior of bands at 1188 and 1172 cm^–1^ may be related to an over-reaction, whose degree depends on the specific morphology of the scanned area. For example, the over-reaction could be caused by nonuniform distribution of reactant and product molecules or by the presence of impurities or defects on the surface. Further investigation is needed to determine the exact cause of this behavior.

### 3.5 Gold Surface-Enhanced Raman Scattering

**Figure 6:**
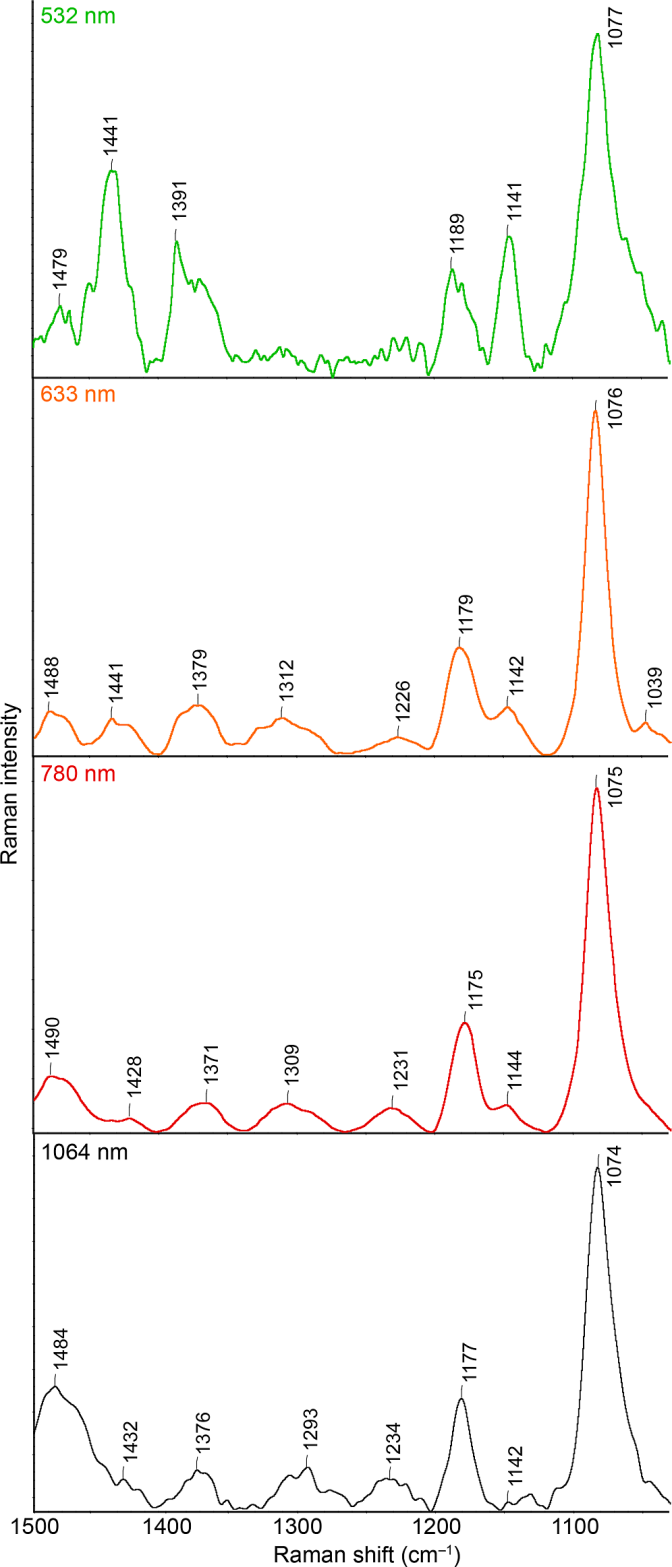
Au-SERS spectra were recorded at different excitation wavelengths. The spectra shown are the result of averaging of 10 (for 532, 633, and 780 nm) and 4 (for 1064 nm) spectra. Spectra are shown in the full-scale mode.

Selected Au-SERS spectra of ABTS are shown in Figure 6. Compared to Ag-SERS spectra, the Au-SERS spectra did not reach such a high intensity, and therefore, for the sake of representativeness, they are shown in the figure in the form of an average from ten or four (in the case of 1064 nm excitation) spectra. In the case of the Au substrate, it was not possible to collect spectra with an excitation wavelength of 455 nm. We assume that this is the case for the reasons given in Chapter 3.3, in short, the plasmon resonance maximum is found at higher wavelengths than this used excitation wavelength.

**Figure 7:**
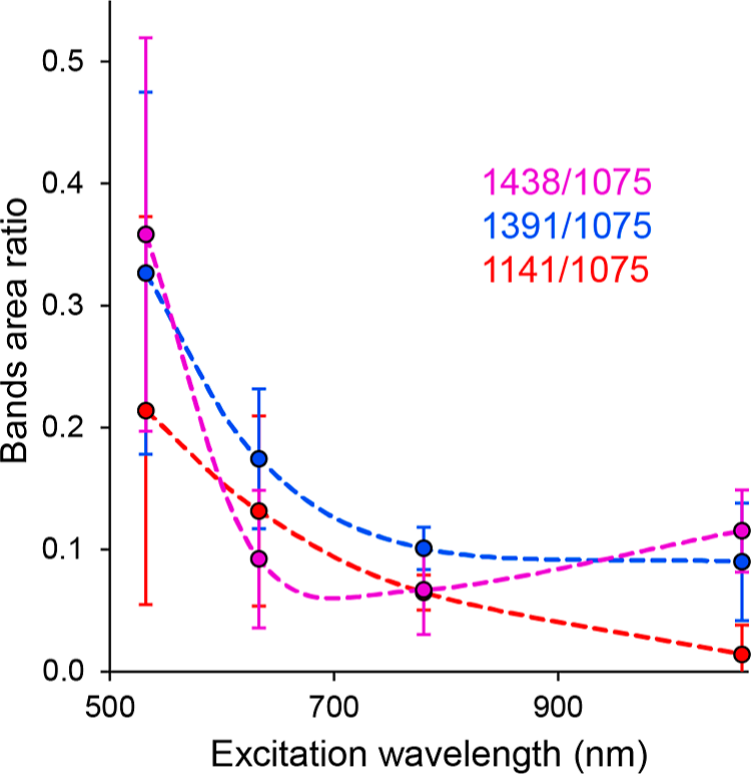
Dependence of the area ratio of the selected Au-SERS bands on the excitation wavelength.

At first glance, it is clear that DMAB bands occur much less frequently in Au-SERS spectra than in the case of Ag. More prominently, these bands (1141, 1391, and 1441 cm^–1^) are observable in the spectrum with 532 nm excitation, just as in the case of Ag, a broadening of some of these bands is observable at this excitation, probably caused by the presence of different ABT species. The mentioned DMAB bands have a significantly lower relative intensity at higher excitation wavelengths than in the case of the same named laser. There is also a shift in the values of the Raman shift of their maximum to lower values. In the case of the band at 1441 cm^–1^, when recorded with a 633 nm excitation, a secondary maximum is discovered at approximately 1430 cm^–1^. This maximum then becomes the main maximum when recording with excitation wavelengths from the NIR region, but its relative intensity continues to decrease. A similar behavior is observed for the band at 1391 cm^–1^. Already in the spectra measured at the 532 nm excitation, two maxima of this band are visible; the second one is located at ca. 1380 cm^–1^. As the value of the excitation wavelength gradually increases, this lower maximum again becomes dominant. We hypothesize that this behavior is also caused by the presence of different ABTS on the Au surface and their different energetic accessibility. Even in the case of Au, it can be stated that the relative intensities of the DMAB bands decrease with an increasing excitation wavelength (Figure 7).

### 3.6 Copper Surface-Enhanced Raman Scattering

**Figure 8:**
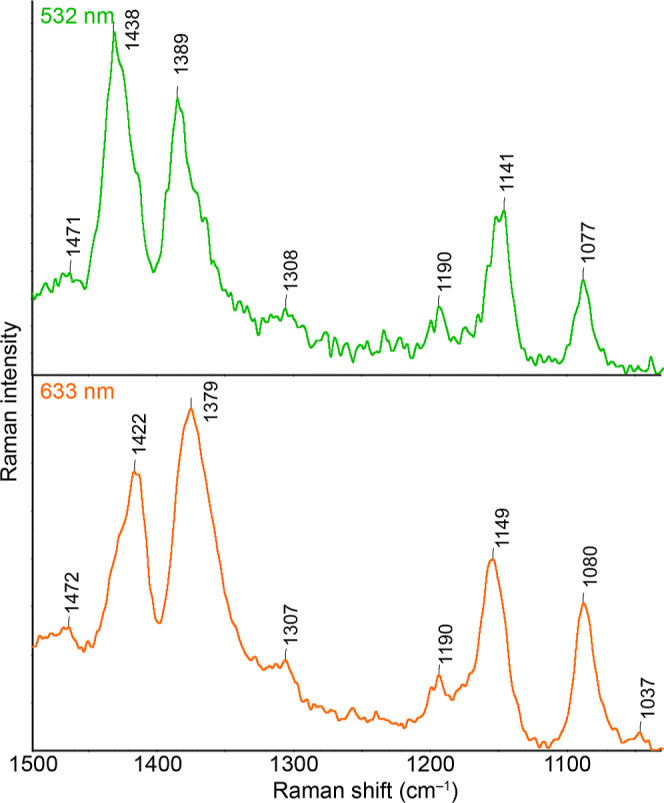
Cu-SERS spectra were recorded at different excitation wavelengths. The spectra shown are the result of averaging of 10 spectra. Spectra are shown in the full-scale mode.

Cu-SERS spectra of ABTS measured with excitation wavelengths of 532 and 633 nm are depicted in Figure 8. As in the case of Au, the spectra shown in the figure are the result of averaging the indicated number of spectra from individual acquisitions. The indicated wavelengths were the only two at which the Cu substrate was shown to be SERS-active. While in the case of the 455 nm excitation, this will probably be due to the lower position of the excitation wavelength relative to the maximum of the plasmon resonance, in the case of excitation wavelengths from the NIR region, we believe that the inactivity of the substrate is associated with a rapid decrease in the intensity of the relevant extinction spectrum, which occurs at shifts to higher wavelengths.

**Figure 9:**
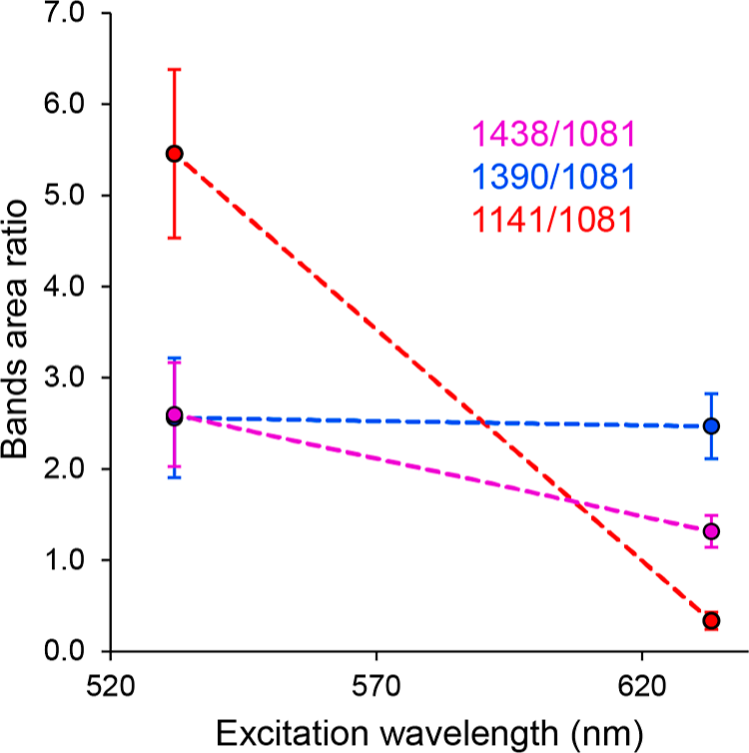
Dependence of the area ratio of the selected Cu-SERS bands on the excitation wavelength.

In both mentioned spectra, there is a clear presence of DMAB bands (1438, 1389, and 1141 cm^–1^ for 532 nm excitation). It is noteworthy that the relative intensity of the DMAB bands is the highest in the case of Cu, compared to those of both Au and Ag. With a shift to a higher excitation wavelength, we can again observe a more pronounced shift of their Raman shift maxima for these bands, specifically to 1422 and 1379 cm^–1^. Interestingly, the band above 1141 cm^–1^ has a value of 1190 cm^–1^ in both cases, while in the Ag and Au cases, it had at least two maxima at several excitation wavelengths, namely, at 1190 and 1170 cm^–1^, the latter being mostly the more intense. The intensity ratios of the individual bands for Cu are listed in Figure 9. While the bands at 1438 and 1141 cm^–1^ decrease with increasing excitation wavelength, the relative intensity of the 1390 cm^–1^ band to the 1081 cm^–1^ band remains almost unchanged.

### 3.7 Time-Varying Behavior on the Ag Surface

**Figure 10:**
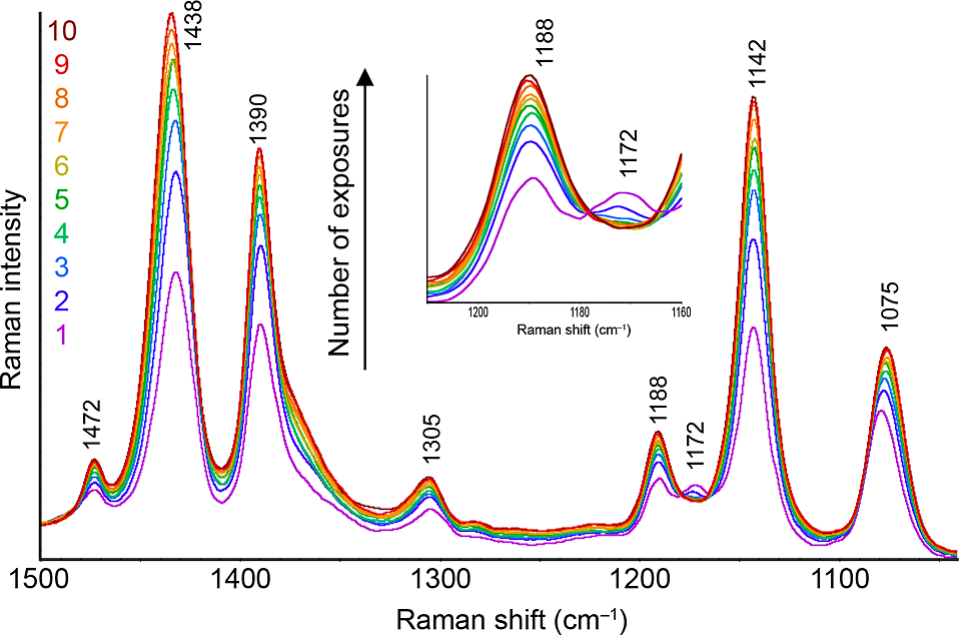
Changes in the spectral profile of ABTS spectra measured with an excitation wavelength of 532 nm during ten consecutive measurements, each lasting 20 s and taken with minimal delays.

Since the results so far clearly led to the fact that a chemical transformation of adsorbed molecules occurs on the surface of the prepared substrates, we focused, similarly to the studies published recently,^34−37^ on the investigation of the kinetics of the ongoing reactions. Only excitation wavelengths of 532 and 633 nm were shown to be SERS-active for all three substrates. For a detailed study, we chose the 532 nm excitation, primarily for its high variability, good intensity, and anomalous behavior of some bands, as can be seen, for example, in Figure 5 for the 1188 and 1172 cm^–1^ bands. In the following experiment, spectra were collected from the selected spot on the substrate ten times consecutively, the changes of which were subsequently re-examined. These spectra are shown in Figure 10. Although the experiment mentioned above was carried out on all three metals, it was not possible to achieve sufficient results in the case of Au and Cu. For this reason, only the results for Ag are presented in this section; the reasons for this outcome will be discussed later.

**Figure 11:**
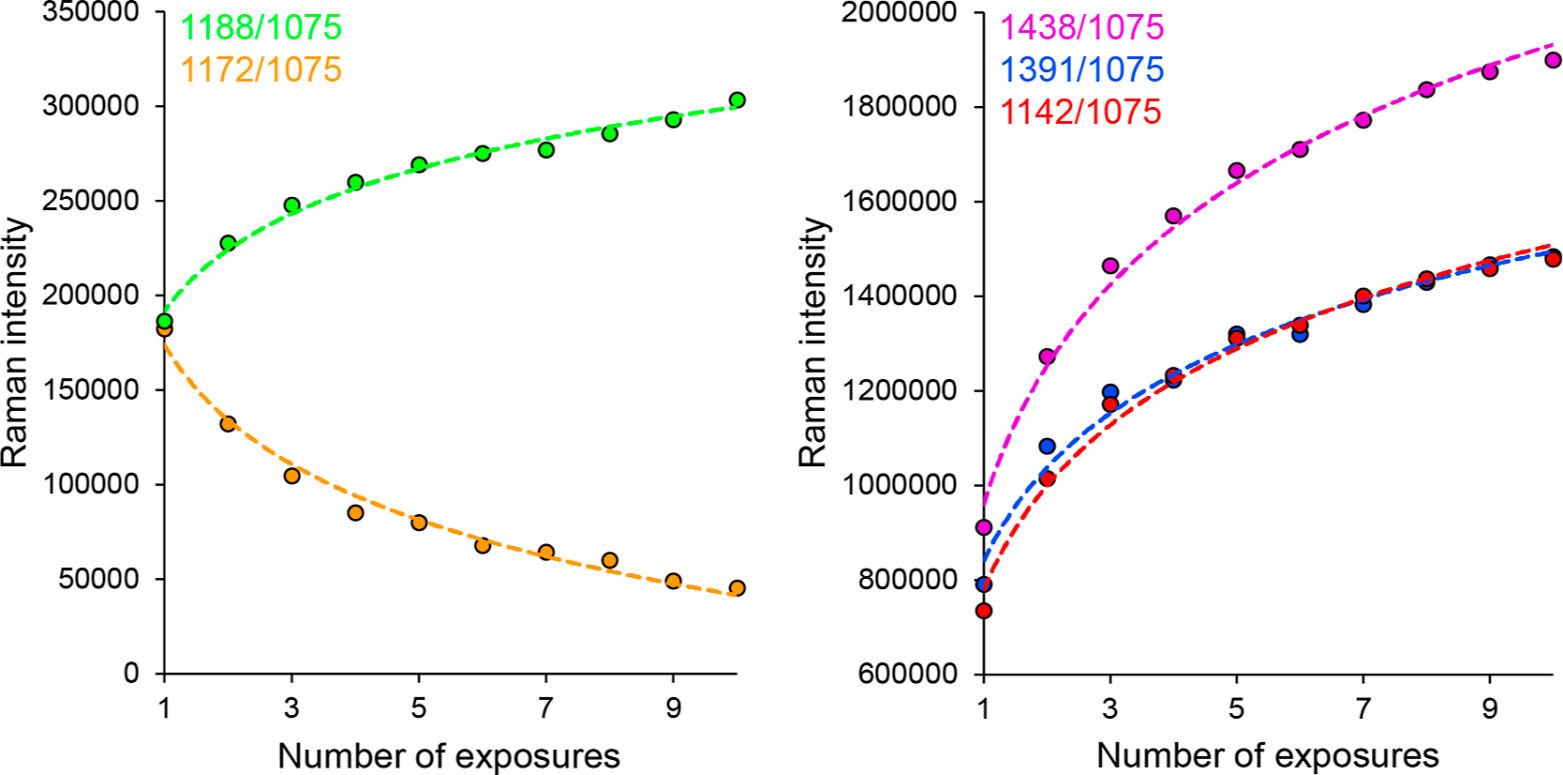
Evolution of absolute intensities of the bands’ areas at 1438, 1391, 1188, 1172, and 1142 cm^–1^ during ten sequential measurements with an excitation wavelength of 532 nm, each lasting 20 s with minimal delays.

At first glance, it is evident that the intensity of the DMAB bands increased during successive measurements. The band at 1075 cm^–1^, which includes both the contribution of bands belonging to DMAB and other ABTS, also underwent certain changes during the measurement. Its intensity also increased, but compared to the previous group of bands, its maximum shifted toward lower values of the Raman shift. This fact corresponds to the DFT predictions as well as to the previous observations. However, the pair of bands at 1188 and 1172 cm^–1^ again behaves most curiously. It is evident that with successive measurements the intensity of the former band increases at the expense of the latter band. The time evolution of the absolute intensities of all discussed bands is shown in Figure 11.

There is an obvious connection between bands 1188 and 1172 cm^–1^; the lying area is only identical at the beginning of the measurement, the area of the band with a higher value of the Raman shift gradually increases, while the area of the lower one decreases. Both dependencies are described very well by logarithmic trend lines. This also applies to the previously discussed trio of bands at 1438, 1391, and 1142 cm^–1^, whose gradual increase in time is also depicted in Figure 11. Due to the considerable similarity of the observed trends with the classical kinetic courses of chemical reactions, we decided to focus on the obtained data from the point of view of chemical kinetics. In the case of the 1172 cm^–1^ band, whose decrease in intensity over time resembles the decrease in reactant concentration during an ongoing chemical reaction, we decided first to determine numerically the order of the reaction from the fit to the experimentally obtained data.

**Figure 12:**
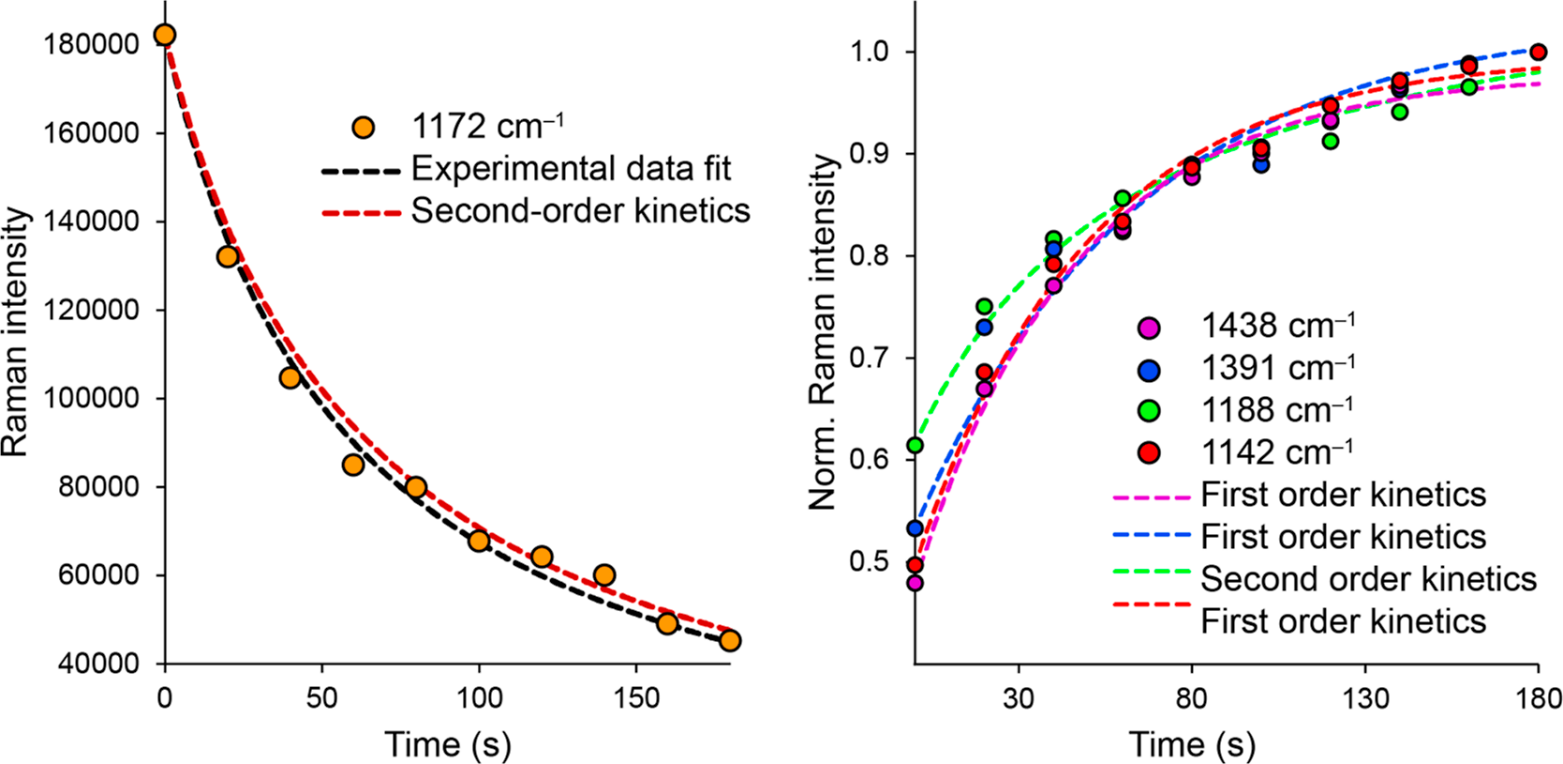
Fits based on kinetic equations are given to experimentally obtained and normalized areas of selected SERS bands.

Please allow us to consider the situation where we declare the first recorded spectrum to be the state at zero time (the time when the irradiation ended) and that the intensity of the observed band is closely related to the substance to which the band belongs. At the same time, we assume that the ongoing reaction is irreversible under the conditions used, which can most probably be stated by considering the observed trends. Ten exposures represent a total time interval of 180 s; individual times are always assigned to the beginning of the acquisition. The obtained results are shown in Figure 12 (black line). From the results of the fit, the value of the reaction order is 2.0067. Thus, we may assume that the ongoing event can be declared as a second-order (Figure 12, red line) reaction within the experimental uncertainties. From the experimental fit, in addition to the information about the order of the reaction, we also extracted the value of the rate constant, *k* = 8.6374 × 10^–8^ SERS intensity^–1^·s^–1^.

After these findings, we focused on the approximate determination of the kinetics of the bands that grow during the sequence, we speak about “product” bands (Figure 12, right). It should be kept in mind that in our case, this term does not necessarily mean a new molecule (in a chemical sense), but, for example, only a different ABTS, differing for example in its conformation, orientation, and/or symmetry concerning the local plane/shape of metal surface, i.e., the term: chemical species is used. In this case, we used normalizing the intensities of these bands (individual bands were always normalized to the highest intensity of the given band in the sequential measurement; that is, to the intensity of the given band in the last spectrum, the relative intensities in the kinetic set are evaluated). This is because the concentration of the resulting “products” is naturally dependent on the current concentration of the “reactants”. Since in our case, we studied the response of the intensity of the bands in the surface-enhanced Raman spectra, the relationship between the intensity of a specific band and the concentration will likely vary considerably depending on which species the band belongs to. The symmetry and surface selection rules themselves may be the reason. In the normalized trends, we, therefore, considered the “concentration” of the reactant at the beginning of the reaction (first spectrum) to be equal to one. Subsequently, we tested the appropriateness of the dependence’s characteristic of the course of various entire orders of chemical reactions, looking for the dependence that would best correspond to the observed experimental trend. It was found that in the case of the 1188 cm^–1^ band, second-order kinetics fit the recorded waveform best (*k*_1188_ = 0.015115 norm. SERS intensity^–1^·s^–1^), while in the case of the other three bands examined, first-order kinetics fit best (*k*_1438_ = 0.021195 s^–1^, *k*_1391_ = 0.015575 s^–1^, *k*_1142_ = 0.020137 s^–1^, and *k*_avrg_ = 0.0190 ± 0.0030 s^–1^). Several explanations are hypothesized. It is possible that the second-order behavior of the 1188 and 1172 cm^–1^ bands describes some kind of precursor reaction that occurs before the formation of DMAB itself. The gradual development of these bands over time provides evidence to support the claim that dimerization is likely the ongoing process. Considering general chemical mechanisms nascent intermediate could be DMHAB, although direct spectral evidence is unfortunately lacking. However, the idea that the reaction from DMHAB to DMAB could follow first-order kinetics (the change only occurs within one molecule) makes this hypothesis at least worth considering.

Furthermore, the dependence of the ratio of the areas of the bands at 1188 and 1172 cm^–1^ to the area of the 1075 cm^–1^ band on the excitation wavelength suggests the possibility of multiple reactions occurring simultaneously in various ratios, some of which are governed by first-order kinetics and some by second-order kinetics. Notably, a significant change in this ratio was observed at a 532 nm excitation wavelength, which may indicate a transition in the relative contribution of the different reaction pathways.

Another possible explanation is found in the very nature of the photochemical reactions taking place. Suppose that such a reaction starts with the excitation of a surface complex, while the wavelength of the laser used must correspond to the range of wavelengths in which the absorption band of the given complex is located. In such a case, a complex is activated, which then most likely relaxes energetically, for example, by transforming into a more energetically favorable complex. The absorption band of the resulting complex would then logically be located at higher wavelengths, which correspond to lower energies. Specifically, for example, in the case of Ag, based on the calculated positions of the absorption bands of the complexes, it can be assumed that the Ag-ABT complexes are activated by the 532 nm excitation radiation. Due to their position, they can then relax into Ag-DMHAB, Ag-DMAB-1, Ag-DMAB-2 and Ag-DMAB-3 complexes, precisely by forming one of these mentioned dimers. This would correspond to second-order kinetics, characteristic of the kinetics of the 1188 cm^–1^ band. Due to their position, already formed dimer complexes can, however, be excited by 532 nm radiation. Theoretically, their transition to less energy-demanding conformations should not be improbable, precisely because of spontaneous transformation, induced by interaction with either photons or plasmons. First-order kinetics would correspond to such events, which is characteristic for the development of the 1438, 1391, and 1142 cm^–1^ bands.

This consideration can also explain the fact that the intensity of the dimer bands is relatively small on the Au surface. If we consider the positions of the absorption bands of the Au-ABT and Au-DMAB-1 complexes, we can state that within the uncertainty of the calculations, their maxima have a very similar value. Thus, it is possible that during the transition to this complex, the incident radiation could at the same time cause a reverse reaction to the Au-ABT complex because the energy required for their excitation should be very similar. Because of probably not only the formation of Au-DMAB-1 but also the formation of other DMAB complexes and Au-DMHAB complexes, the characteristic manifestations of these species can still be observed in the spectra. Together with the fact that there was a high noise value in the obtained experimental Au-SERS spectra, these considerations could explain why it was not possible to record kinetics similar to those in the case of Ag. If we allow ourselves to declare the band at 1190 cm^–1^ as a band belonging to dimer complexes, while the bands at 1170 cm^–1^ belong to monomeric ABTS, we can probably also explain the behavior on Cu. Here, from the very beginning, we observe that the discussed band has a maximum at 1190 cm^–1^. Therefore, it is possible that the dimerization itself takes place very easily here and that a vast majority of molecules are already converted during the first exposure. The resulting Cu-DMAB complexes can then be so energetically efficient that mutual transitions between them no longer occur.

## 4 Conclusions

In this study, we investigated the laser-induced reactions of various surface complexes of 4-aminobenzenethiol on Ag, Au, and Cu surfaces. By employing different excitation wavelengths, we observed distinct behaviors of these molecule species on the three plasmonic substrates. Through the implementation of DFT calculations, we established that the observed behavior is primarily attributed to the action of chemical enhancement mechanisms.

As a consequence of the interaction between 4-aminobenzenethiol molecules and plasmonic surfaces, surface complexes were formed, exhibiting absorption bands significantly red-shifted compared to those of the pure substance, extending into the visible and near-infrared regions. Excitation wavelengths from these regions enabled the induction of photochemical transformations, with the nature of the transformations varying significantly based on the excitation wavelength and the plasmonic metal employed.

Furthermore, the resonance with the absorption transitions of these complexes played a key role in enhancing the intensity of SERS, enabling the examination of detailed information about the ongoing processes. An exemplary case is a kinetic study conducted on the Ag surface, where a comprehensive investigation of the time evolution of individual bands allowed us to identify processes governed by either first- or second-order kinetics. While the dimerization process of 4-aminobenzenethiol molecules was attributed to second-order kinetics, the first-order kinetics likely originated from the transformation processes of individual molecules due to their interaction with photons or plasmons, such as the reorientation of dimer molecules relative to the surface.

It is crucial to recognize that the behavior of the molecules is largely determined by the position and variability of the band between 1170 and 1190 cm^–1^. The position of the former corresponds to the molecules in the monomer state, while the position of the latter corresponds to the dimerized ones. This band provides a direct means of observing the dimerization kinetics of the molecules. Comparing these bands on Ag, Au, and Cu surfaces at a 532 nm excitation wavelength, we infer that laser-induced dimerization occurs most rapidly on the Cu surface, followed by Ag, and least on Au.

## Supporting Information Available

SEM images; TEM images; DFT associated content; calculations’ results; and vibrational assignments (PDF)

## Abbreviations

4-ABT: 4-aminobenzenethiol
4,4′-DMAB: 4,4′-dimercaptoazobenzene
ABTS: aminobenzenethiol species
CT: charge transfer
DFT: density functional theory
DMHAB: dimercaptohydroazobenzene
LSPR: localized surface plasmon resonance
NBT: nitrobenzenethiol
NIR: near infrared
EDS: energy dispersive spectroscopy
SEM: scanning electron microscopy
SERS: surface-enhanced Raman spectroscopy
SPR: surface plasmon resonance
TEM: transmission electron microscopy
Vis: visible spectroscopy
