# Supplementary material for: Laser-Induced Reactions of 4-Aminobenzenthiol Species Adsorbed on Ag, Au, and Cu Plasmonic Structures Followed by SERS Spectroscopy. The Role of Substrate and Excitation Energy – Surface-Complex Photochemistry and Plasmonic Catalysis
Source: ACS Omega. 2024 Jan 25;9(5):6005–17. doi: 10.1021/acsomega.4c00121 (PMC10851236; doi:10.1021/acsomega.4c00121)
Supplement: Supplementary file 1 — ao4c00121_si_001.pdf [file ao4c00121_si_001.pdf]

Supplementary Information

Laser-Induced Reactions of 4-Aminobenzenthiol Species Adsorbed on Ag, Au, and Cu Plasmonic Structures followed by SERS spectroscopy. The Role of Substrate and Excitation Energy – Surface-Complex Photochemistry and Plasmonic Catalysis

Ivan Kopal[1,2], Marie Švecová[3], Vojtěch Jeřábek[1], David Palounek[1,2], Tereza Čapková[4], Alena Michalcová[5], Ladislav Lapčák[1,6], Pavel Matějka[1], Marcela Dendisová[1]*

[1] Department of Physical Chemistry, University of Chemistry and Technology Prague, Technická 5, 166 28 Prague 6, Czech Republic

[2] Institute of Photonics and Electronics, Czech Academy of Sciences, Chaberská 1014/57, 182 00 Prague 8, Czech Republic

[3] Department of Analytical Chemistry, University of Chemistry and Technology Prague, Technická 5, 166 28 Prague 6, Czech Republic

[4] Centre of Polymer Systems, University Institute, Tomas Bata University in Zlín, Třída Tomáše Bati 5678, 760 01 Zlín, Czech Republic

[5] Department of Metals and Corrosion Engineering, University of Chemistry and Technology Prague, Technická 5, 166 28 Prague 6, Czech Republic

[6] Central Laboratories, University of Chemistry and Technology, Technická 5, 166 28 Prague, Czech Republic

* Corresponding author: tel.: + 420 220 443 694, e-mail: kopali@vscht.cz

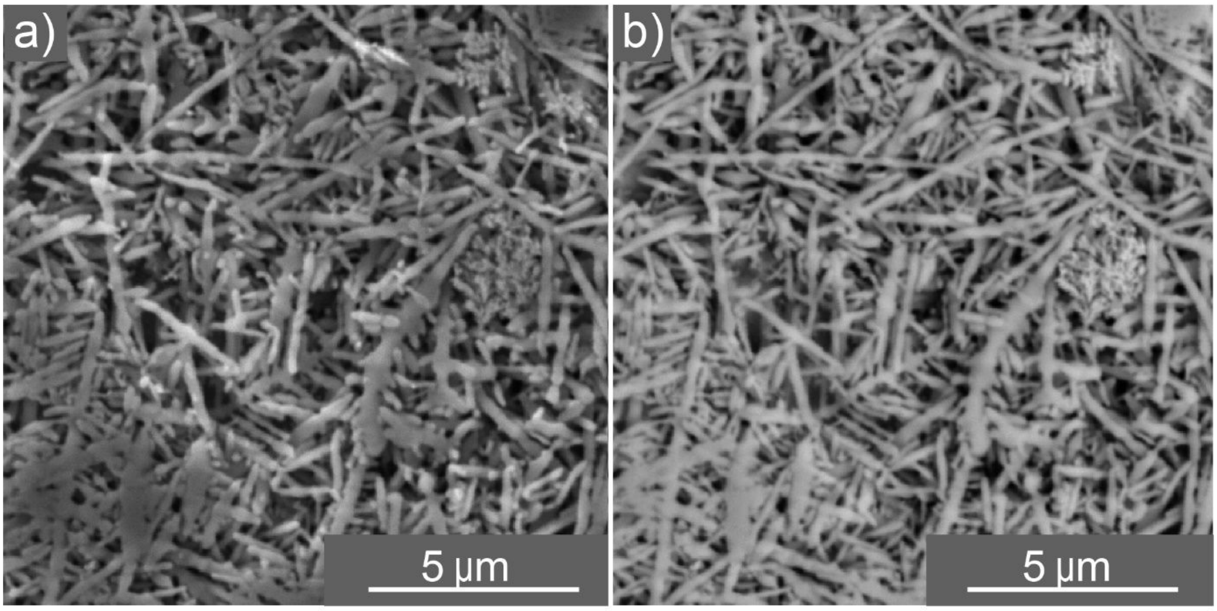

Figure S1. SEM image of a) secondary electrons and b) backscattered electrons on the Ag surface.

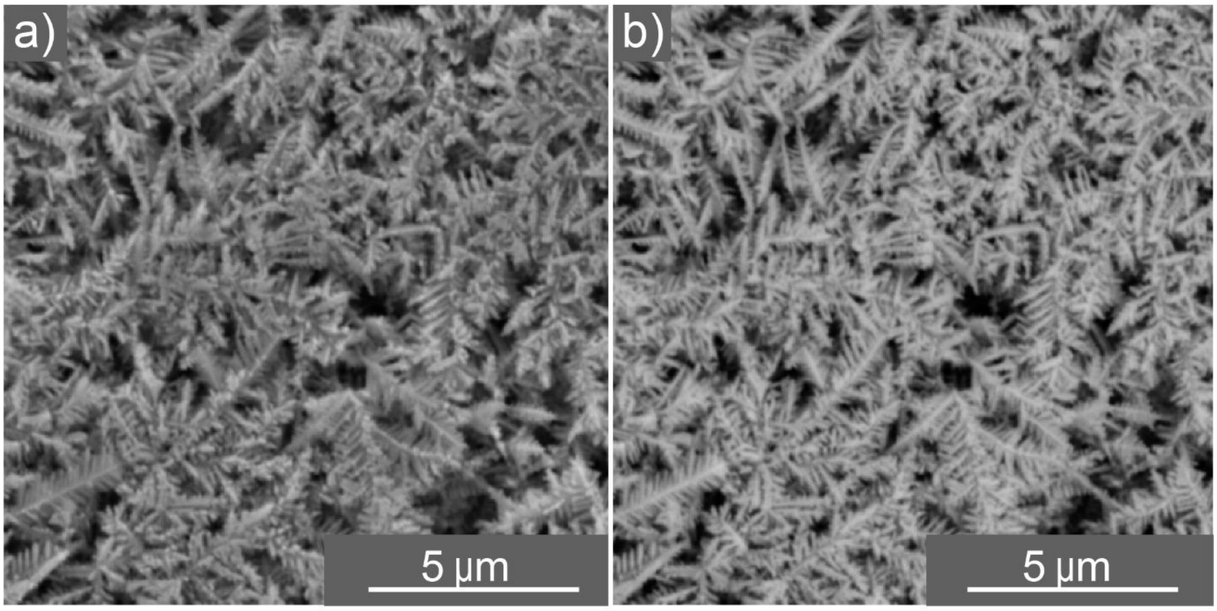

Figure S2. SEM image of a) secondary electrons and b) backscattered electrons on the Au surface.

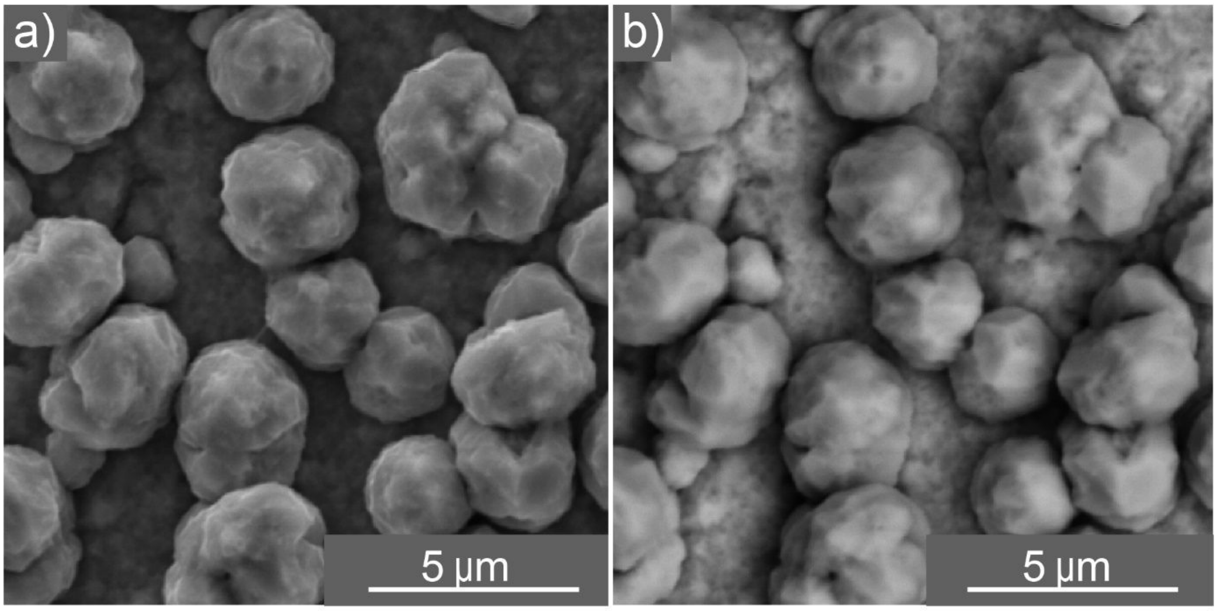

Figure S3. SEM image of a) secondary electrons and b) backscattered electrons on the Cu surface.

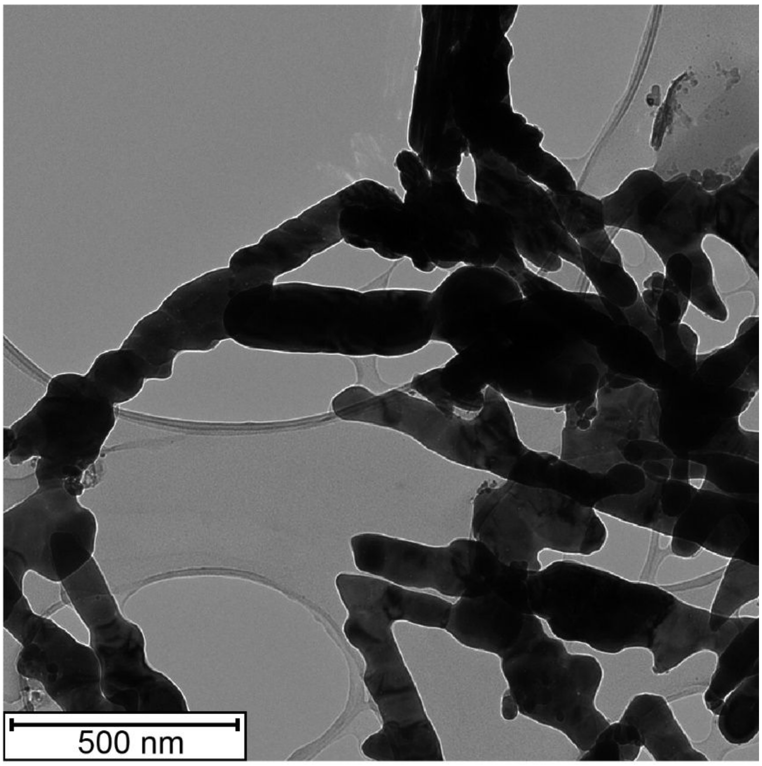

Figure S4. TEM image of Ag nano-structures.

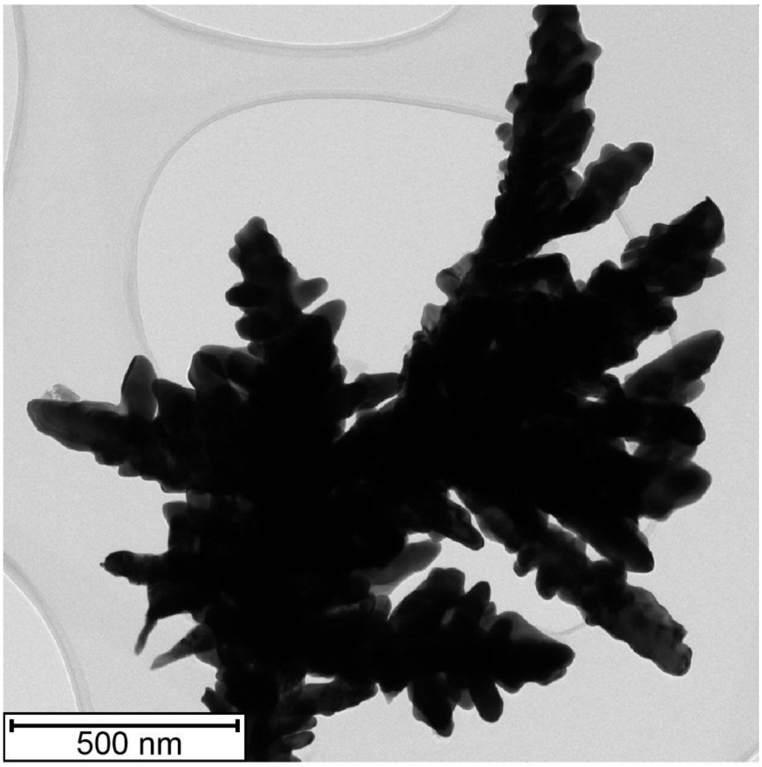

Figure S5. TEM image of Au nano-structures.

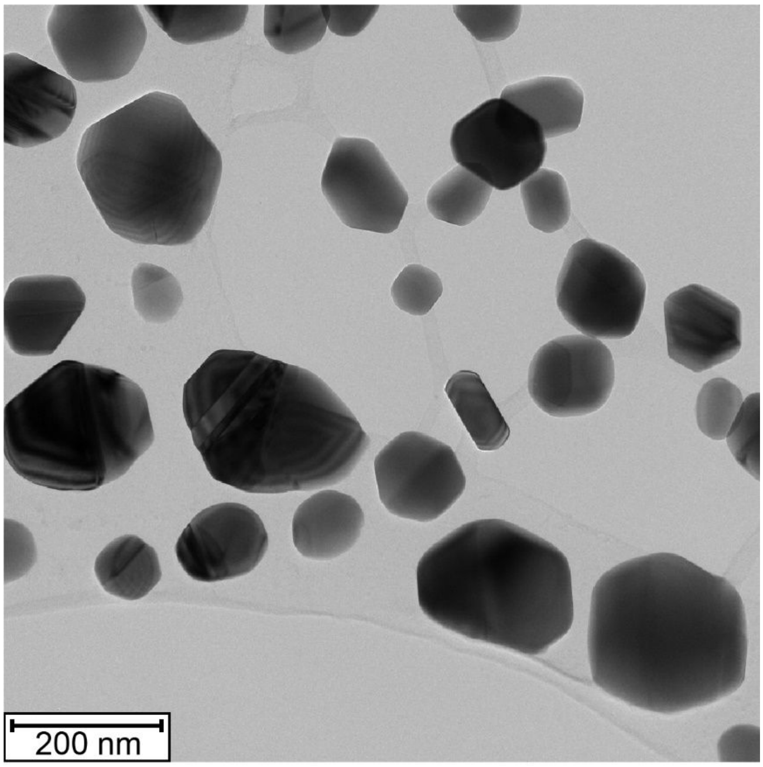

Figure S6. TEM image of Cu nano-structures.

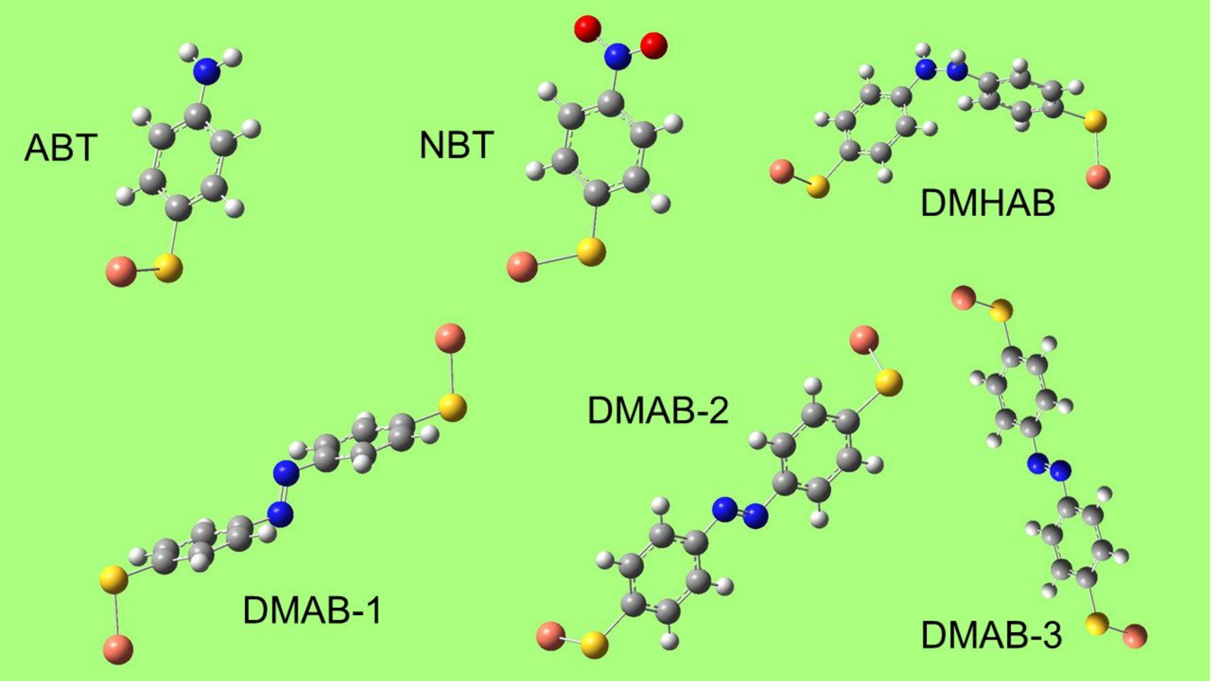

Figure S7. Representation of "metal-molecule" complexes used for DFT calculations.

Table S1. Overview of absorption band maxima of individual "metal-molecule" complexes.

		Wavelength (nm)					
		Molecule					
		ABT	NBT	DMHAB	DMAB-1	DMAB-2	DMAB-3
Metal	Ag	598	444	632	568	657	621
	Au	541	442	567	546	661	666
	Cu	580	419	577	584	597	615

Table S2. Maxima of Ag-complexes Raman bands according to DFT calculation and assignment of vibrational modes.

Raman shift (cm^{-1})						
ABT	NBT	DMHAB	DMAB-1	DMAB-2	DMAB-3	Assignment of the vibrational modes
1064	1060	1064	1065	1056	1056	ν C–S
	1098			1099	1099	ν C–N + C–H
			1136	1142	1142	ν C–N + C–H
1186	1180	1183	1183	1188	1187	δ_{ip} C–H
	1241	1245				ν C–N + δ_{ip} C–H
1298				1292	1292	ν C–N + δ_{ip} C–H
1335						ν_{ar} C–C + δ_{rock} N–H + δ_{ip} C–H
				1331	1332	ν_{ar} C–C + δ_{ip} C–H
		1315				ν N–N + ν_{ar} C–C + δ_{ip} C–H
		1352				ν N–N + ν_{ar} C–C + δ_{ip} C–H
	1381					ν_{ar} C–C + ν_{as} N–O + δ_{ip} C–H
				1380	1381	ν N=N + ν_{ar} C–C + δ_{ip} C–H
			1441	1419	1421	ν N=N + ν_{ar} C–C + δ_{ip} C–H
1484	1460					ν C–N
		1468				δ N–H
				1557	1559	ν_{ar} C–C + δ_{ip} C–H
		1576				ν_{ar} C–C + δ N–H + δ_{ip} C–H
	1580		1589	1585	1586	ν_{ar} C–C + δ_{ip} C–H
1607						ν_{ar} C–C + ν C–N + δ_{sci} N–H + δ_{ip} C–H
		1598				ν_{ar} C–C + δ N–H + δ_{ip} C–H

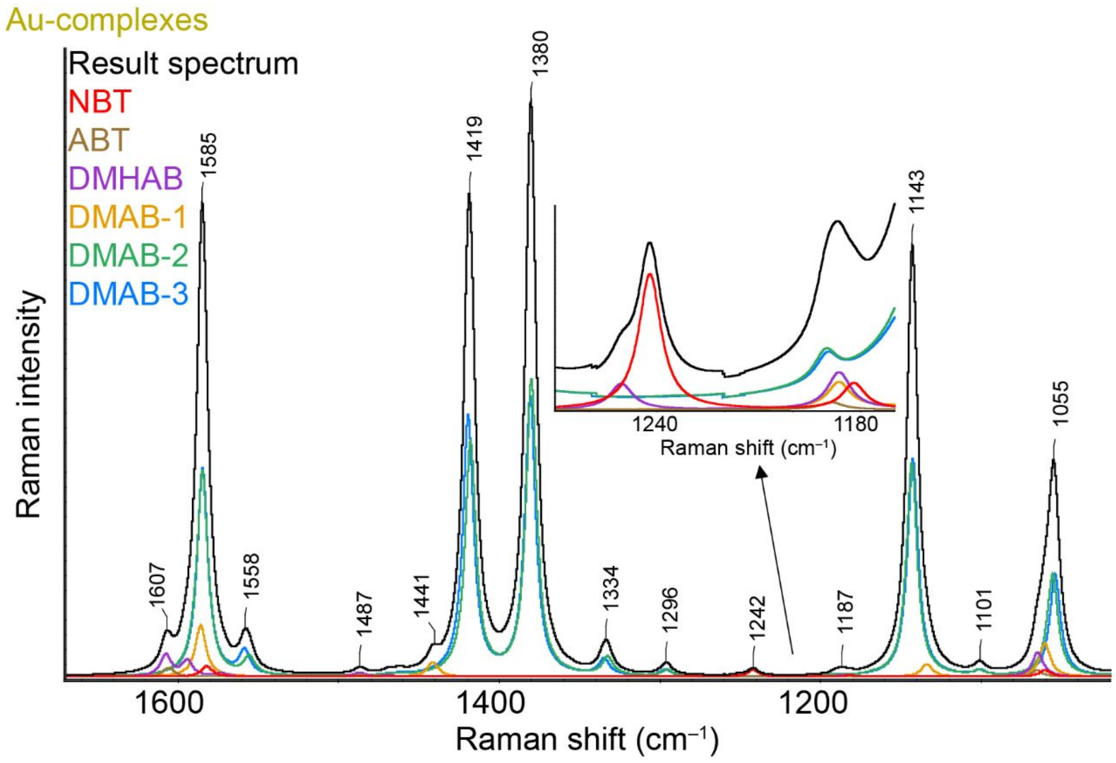

Figure S8. DFT calculated Raman spectra of the theoretically formed "Au-ABTS" complexes and their theoretical sum spectrum (black line).

Table S3. Maxima of Au-complexes Raman bands according to DFT calculation and assignment of vibrational modes.

Raman shift (cm^{-1})						
ABT	NBT	DMHAB	DMAB-1	DMAB-2	DMAB-3	Assignment of the vibrational modes
1065	1061	1065	1061	1056	1054	ν C–S
	1096			1101	1011	ν C–N + C–H
			1134	1143	1143	ν C–N + C–H
1189	1182	1186	1186	1190	1189	δ_{ip} C–H
	1242	1251				ν C–N + δ_{ip} C–H
1309				1296	1297	ν C–N + δ_{ip} C–H
1345						ν_{ar} C–C + δ_{rock} N–H + δ_{ip} C–H
				1333	1335	ν_{ar} C–C + δ_{ip} C–H
		1322				ν N–N + ν_{ar} C–C + δ_{ip} C–H
		1356				ν N–N + ν_{ar} C–C + δ_{ip} C–H
	1381					ν_{ar} C–C + ν_{as} N–O + δ_{ip} C–H
				1380	1381	ν N=N + ν_{ar} C–C + δ_{ip} C–H
			1441	1418	1419	ν N=N + ν_{ar} C–C + δ_{ip} C–H
		1469				δ N–H
1487	1461	1487				ν C–N
				1556	1559	ν_{ar} C–C + δ_{ip} C–H
		1573				ν_{ar} C–C + δ N–H + δ_{ip} C–H
	1582		1586	1585	1585	ν_{ar} C–C + δ_{ip} C–H
1606						ν_{ar} C–C + ν C–N + δ_{sci} N–H + δ_{ip} C–H
		1595				ν_{ar} C–C + δ N–H + δ_{ip} C–H

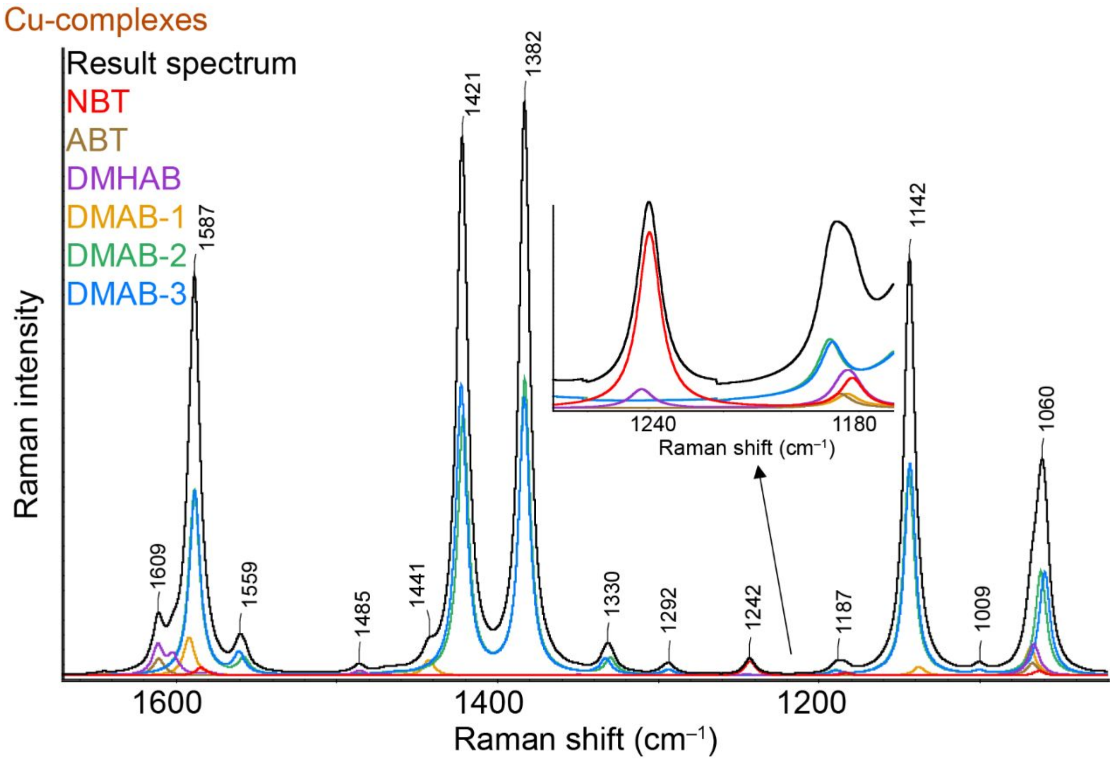

Figure S9. DFT calculated Raman spectra of the theoretically formed "Cu-ABTS" complexes and their theoretical sum spectrum (black line).

Table S4. Maxima of Cu-complexes Raman bands according to DFT calculation and assignment of vibrational modes.

Raman shift (cm^{-1})						
ABT	NBT	DMHAB	DMAB-1	DMAB-2	DMAB-3	Assignment of the vibrational modes
1066	1062	1065	1067	1061	1058	ν C–S
	1101			1099	1099	ν C–N + C–H
			1137	1143	1142	ν C–N + C–H
1186	1182	1183	1184	1189	1188	δ_{ip} C–H
	1242	1244				ν C–N + δ_{ip} C–H
1297				1292	1293	ν C–N + δ_{ip} C–H
1335						ν_{ar} C–C + δ_{rock} N–H + δ_{ip} C–H
				1329	1332	ν_{ar} C–C + δ_{ip} C–H
		1315				ν N–N + ν_{ar} C–C + δ_{ip} C–H
		1348				ν N–N + ν_{ar} C–C + δ_{ip} C–H
	1383					ν_{ar} C–C + ν_{as} N–O + δ_{ip} C–H
				1382	1382	ν N=N + ν_{ar} C–C + δ_{ip} C–H
			1441	1420	1421	ν N=N + ν_{ar} C–C + δ_{ip} C–H
		1464				δ N–H
1485	1461	1485				ν C–N
				1557	1560	ν_{ar} C–C + δ_{ip} C–H
		1578				ν_{ar} C–C + δ N–H + δ_{ip} C–H
	1583		1591	1587	1587	ν_{ar} C–C + δ_{ip} C–H
1610						ν_{ar} C–C + ν C–N + δ_{sci} N–H + δ_{ip} C–H
		1601				ν_{ar} C–C + δ N–H + δ_{ip} C–H
